# Effect of Acute Normobaric Hypoxia Exposure on Executive Functions among Young Physically Active Males

**DOI:** 10.3390/jcm10081560

**Published:** 2021-04-08

**Authors:** Maciej Chroboczek, Maciej Kostrzewa, Katarzyna Micielska, Tomasz Grzywacz, Radosław Laskowski

**Affiliations:** 1 Department of Physiology and Biochemistry, Gdansk University of Physical Education and Sport, 80-336 Gdansk, Poland; 2 Department of Sports Training, Jerzy Kukuczka Academy of Physical Education in Katowice, 40-065 Katowice, Poland; m.kostrzewa@awf.katowice.pl; 3 Department of Physical Education and Lifelong Sports, Poznań University of Physical Education, 61-871 Poznań, Poland; micielska@awf.poznan.pl; 4 Department of Sport, Institute of Physical Education, Kazimierz Wielki University, 85-064 Bydgoszcz, Poland; tomgrzyw@ukw.edu.pl; 5 Laboratory of Exercise Biochemistry and Neuroendocrinology, Department of Sports Neuroscience, Advanced Research Initiative for Human High Performance (ARIHHP), Faculty of Health and Sports Sciences, University of Tsukuba, Ibaraki 305-8577, Japan

**Keywords:** cognitive function, physical exercise, altitude

## Abstract

Background: On the one hand, hypoxic exposure may result in progressive brain metabolism disturbance, causing subsequent cognitive impairments. On the other hand, it might also enhance neurogenesis and brain vascularization as well as accelerate cerebral blood flow, leading to cognitive function improvement. The aim of this study was to investigate whether progressive stages of normobaric hypoxia (NH) (FIO_2_ = 13%, FIO_2_ = 12%, and FIO_2_ = 11%) differentially affect post-exposure cognitive performance. Methods: Fifteen physically active men (age = 23.1 ± 2.1) participated in the study. The Stroop test (ST) was applied to assess cognitive function. To generate NH conditions, a hypoxic normobaric air generator was used. Results: We observed an executive function impairment (“naming” interference *p* < 0.05) after NH exposure (FIO_2_ = 13%). After exposure at FIO_2_ = 12% and FIO_2_ = 11%, no changes were observed in the Stroop test. Also, changes in SpO_2_ during subsequent NH exposure were observed. Conclusions: The current investigation shows that executive functions deteriorate after acute NH exposure and this post-exposure deterioration is not proportional to the normobaric hypoxia stages among young physically active males.

## 1. Introduction

Cognitive functions are highly dependent on adequate oxygen delivery to the brain [1]. Elevated brain activity during cognitive processing causes a rise in energy demand, leading to an increase in cerebral blood flow (CBF) [2]. It is assumed that the energy demand of neuronal tissue increases by 15% during tasks that require cognitive functioning [3,4,5,6]. Therefore, disturbances in cerebral aerobic metabolism, caused by hypoxia, could manifest as a cognitive function impairment [2].

Cognitive impairment can negatively affect residents or workers staying at high altitudes, e.g., mountain guides, militaries, athletes at altitude training camps, mountaineers or skiers, as well as exposed-to-hypoxia aircraft pilots or flight personnel [7,8,9]. Moreover, cognitive decline is observed commonly among patients with severe forms of hypoxic–ischemic brain injury such as stroke, sleep apnea, or chronic obstructive pulmonary disease (COPD) [10,11,12,13,14,15,16,17]. Additionally, reduced vascularization and blood flow in aging also diminish the ability to supply oxygen to the brain, leading to cognitive function impairment [18,19,20,21].

Hypoxic exposure, therefore, impairs memory, color vision, reaction time, and executive functions [22]. The deterioration of cognitive abilities seems to be also dependent on the magnitude of hypoxia and exposure time [22]. It also appears that hypoxic exposure response is largely dependent on hypoxic mode and protocol (hypobaric/normobaric, intermittent/continuous), the participants’ age, fitness level, and health status, cognitive task type, post-cognitive test timing, and other confounding factors [22,23,24,25,26]. Numerous human studies have revealed that normobaric hypoxia at various exposure times (from 16 to 30 min) and at different simulated altitudes have a detrimental influence on reaction time and error rate during cognitive performance [27,28]. Nevertheless, Pavlicek et al. (2005) and Taylor et al. (2015) did not observe changes in human cognition (word fluency, word association task) after 30–45 min exposure to simulated altitudes (2440 m–4500 m a.s.l.) [29,30]. 

However, mild hypoxic exposure can also have a neuroprotective effect in improving cognitive performance; therefore, a right dosage or time of exposure/measurement could be a key factor [31,32]. Moreover, a combination of mild hypoxia and aerobic training seems to enhance cognitive function among the elderly [33]. Furthermore, some NH protocols show enhancement of cognitive functions; for example, intermittent hypoxia exerts a beneficial impact on protective mechanisms [34]. In a study conducted by Loprinzi et al. (2019), a positive effect of acute normobaric hypoxia on memory interference was observed [35]. Although hypoxia may lead to progressive disturbance in brain metabolism, causing subsequent cognitive impairments, it might also induce the synthesis of catecholamines and neurotrophins such as brain-derived neurotrophic factor (BDNF) or vascular endothelial growth factor (VEGF) as well as accelerate CBF, positively affecting neurogenesis and brain vascularization [22,30,36]. Hypoxia can induce the release of BDNF, relevant for memory formation and potentiation; therefore, a positive effect might be expected [37].

The aim of this study was to investigate whether cognitive abilities among young physically active males could be facilitated after normobaric hypoxia exposure. We hypothesized that progressive stages of normobaric hypoxia (FIO_2_ = 13%; FIO_2_ = 12% and FIO_2_ = 11%) affect human cognition differently.

## 2. Materials and Methods

### 2.1. Participants

Twenty-four healthy, non-obese young adults were enrolled to the experiment. At the beginning, four subjects participated in Experiment 1 (a pilot study), and after its completion, the experimental group was enlarged to up to 20 participants taking part in the main study—Experiment 2. At the end, 15 physically active men finished the main experiment (5 of them did not complete it due to several reasons, e.g., cold, absence, or personal reasons). All participants were Polish native speakers. Before the start of the experiment, they were introduced to the procedures to which they had volunteered. Exclusion criteria included a history of alpine expedition, dyslexia, daltonism, and blurred vision. All participants were university students and were physically active. The participants did not have any medical contraindications. No participant stated a history of neurological, psychiatric, or respiratory disorders or had a disease that required medical care. Additionally, they were required to refrain from consumption of caffeine 24 h prior to the testing session. All participants gave their written consent after they had been informed about the purpose of the study and the procedures. The study was approved by the local Ethics Committee and the Bioethical Committee of the Regional Medical Society (KB-9/16) according to the Helsinki Declaration. Detailed anthropometric characteristics of the participants are presented in Table 1.

### 2.2. Measures

#### 2.2.1. Anthropometric Measurements 

To measure body height in a standing position, an anthropometer from a GPM measuring set—Skinfold Caliper User’s Manual (Poland)—was used. Body mass and body composition: body fat (FAT) and fat-free mass (FFM), were measured using the TBF-300 Tanita Body Fat Monitor/Scale Analyzer (Japan) with the use of the bioelectrical impedance method. The body mass index was also used to assess overall body build: relative body mass (BMI) [kg·m^−2^]. The participants were asked to arrive at the laboratory fasted, with voided bladders and bowels [38].

#### 2.2.2. Normobaric Hypoxia (NH)

The GO2Altitude ERA II Hypoxic/hyperoxic air generator from Biomedtech (Australia) was used to create the appropriate hypoxic conditions during the tests. The proposed altitudes (a.s.l.) were simulated by reducing the oxygen content of the inspiratory mixture according to the recommendations of the manufacturer Biomedtech Australia Pty. Ltd. Biomedical Research and Development described in GO2 Altitude ERA II Hypoxicator System Operational Manual and in [39]. To produce a hypoxic mixture constituting a simulation of altitude at 3500 m, the oxygen level in the mixture (FIO_2_ = 13%) was used, at 4500 m (FIO_2_ = 12%) and 5500 m (FIO_2_ = 11%), respectively. The participants were not aware of the simulated altitude. When performing tests in normoxia (NOR), the participants also wore masks connected to the generator and pulse oximeters simulating hypoxic conditions; however, at that time, the air generator produced a breathing mixture occurring naturally at sea level. Additionally, the oxygen saturation (SpO_2_) was measured using a BEURER PO60 pulse oximeter during the whole experimental procedure.

### 2.3. Cognitive Functions

#### 2.3.1. Stroop Interference Test (ST)

To measure cognitive control, the computer version of the ST test from the Vienna Test System database was used. The first part involves giving “names” of colors. Part two is about “reading” color names. The third part requires giving the name of the font color with which each word was written instead of reading the written word. For example, the “blue” stimulus should be reacted with the word “red,” suppressing the natural tendency to read “blue.” Such a task requires constant control and suppression of a natural automatic response in favor of a task consciously managed and subordinated to the rules. The result usually contains several elements, including the time of each test, the difference between the time of the first and the third test and the number of errors in the third test [40].

#### 2.3.2. Design and Procedures

All participants were asked to refrain from exercise and the consumption of alcohol and caffeine for at least 24 h prior to each experiment to control the outside factors that could affect cardiovascular and executive functions. The participants underwent a familiarization to all the equipment needed to conduct the experiment as well as anthropometric examinations. The experimental protocol was performed on five non-consecutive days (familiarization on day one and each altitude on a different day with a one-week break in between to avoid the learning effect). All hypoxic measurements were determined in four different conditions: normoxia and FIO_2_ = 13%, FIO_2_ = 12%, and FIO_2_ = 11%, which correspond to simulated altitudes of 3500 m, 4500 m, and 5500 m, respectively. Experiment consisted of cognitive tests and gas mixture breathing. The participants underwent cognitive testing before and immediately after a 30 min acute exposure to the mentioned conditions. 

A single blind protocol was used (the participants did not know under what conditions or on which day of testing they would be evaluated). During the testing, SpO_2_ was monitored continuously using a BEURER PO60 pulse oximeter. Before performing the tests, the subjects were examined by a medical doctor.

#### 2.3.3. Statistical Analysis

All data were collected to create a single data sheet for statistical analysis. Microsoft Excel v.10.0 for Windows was used for initial archiving and statistical processing of results. Statistical analysis was performed using the tools of GraphPad Prism 7. Arithmetic means, standard deviation, and significance levels of differences between means were calculated. Then, descriptive statistics were used, where a non-parametric paired version of the Student’s *t*-test was used to examine the distribution of each variable. Then, we used two-way analysis of variance (ANOVA), with repeated measures, to investigate the significance of differences between groups and time. Significant main effects were further analyzed using the Bonferroni post hoc test. Significance for all analyses was assumed at *p* < 0.05.

## 3. Results

### 3.1. Cognitive Functions

#### Experiment 1

**Figure 1:**
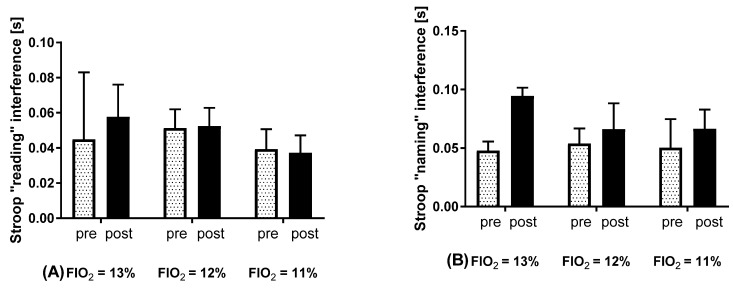
Effect of acute normobaric hypoxia at various simulated altitudes (a.s.l.) on post-exposure interference values in reading (**A**) and in naming (**B**). Values are means. Error bars indicate ± SEM (standard error of the mean).

Deterioration of ST results at all implemented oxygen concentrations was observed, although these changes did not correspond to the increase in simulated altitude values. Noticeable changes were observed at the simulated altitude of 3500 m a.s.l. The analysis revealed no statistical differences either in the reading interference values (interaction F(2, 12) = 0.1374; *p* = 0.8729; time F(2, 12) = 0.5171; *p* = 0.6090) or in the naming interference values (interaction F(2, 12) = 1.647; *p* = 0.2334; time F(2, 12) = 0.8909; *p* = 0.4358), but a noticeable trend in naming was noticed (Figure 1).

#### Experiment 2

**Figure 2:**
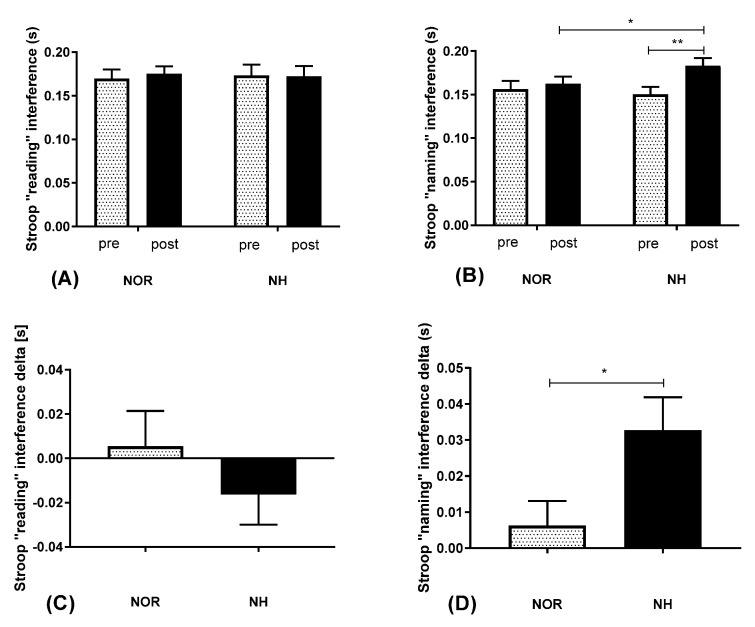
Effect of normoxia and acute normobaric hypoxia at FIO_2_ = 13% on post-exposure interference values in reading (**A**) and in naming (**B**). Subsections (**C**,**D**) represent their deltas. Values are means. Error bars indicate SEM (standard error of the mean). * *p* < 0.05; ** *p* < 0.01. NOR—normoxia; NH—normobaric hypoxia.

As a result of Experiment 1 tests, we enlarged our group and performed cognitive tests at simulated 3500 m a.s.l. (FIO_2_ = 13%). There were no statistical differences in reading interference values (interaction F(1, 28) = 0.007749; *p* = 0.7828; time F(1, 28) = 0.03697; *p* = 0.8489) (Figure 2A). However, statistically significant changes were observed in naming interference values (interaction F(1, 28) = 5.404; *p* = 0.0276; time F(1, 28) = 11.73; *p* = 0.0019; *η*2 = 0.16765) (Figure 2B). Next, contrast analysis between NOR (post–pre) versus NH (post–pre) was performed. The delta reading interference was not significant: *t* = 0.9252; *p* = 0.3705 (Figure 2C). Nevertheless, the delta in naming interference was significantly different between groups: *t* = 2.392; *p* = 0.0314; Cohen’s *d* = 0.878572, paired *t*-test (Figure 2D).

### 3.2. Blood Saturation

#### Experiment 1

**Figure 3:**
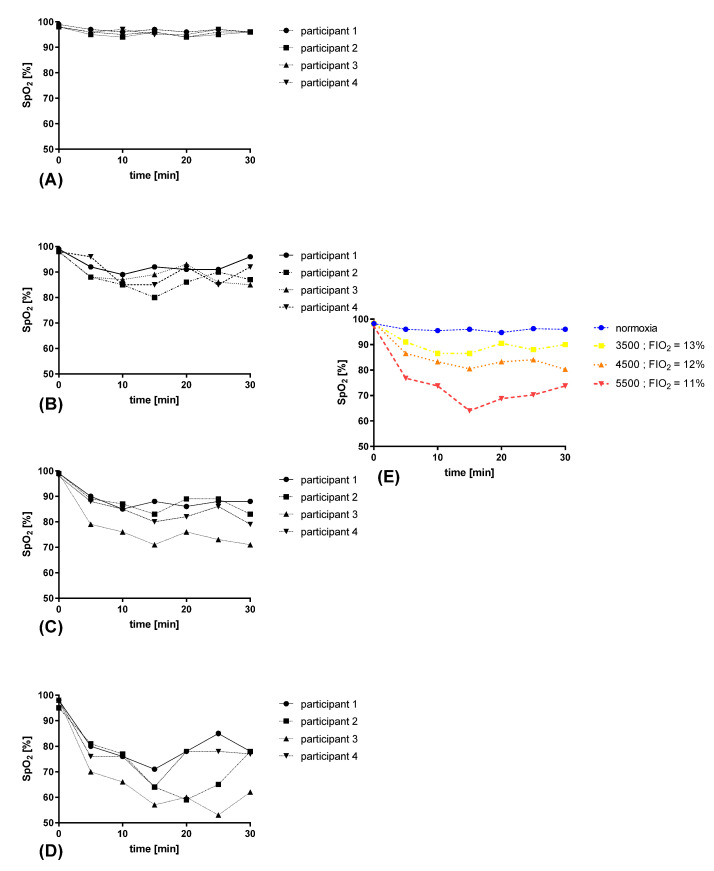
Effect of acute normobaric hypoxia exposure at various simulated altitudes (a.s.l.) on blood saturation: (**A**) saturation under normoxia; (**B**–**D**) saturation at FIO_2_ = 13%, FIO_2_ = 12%, and FIO_2_ = 11%, respectively; (**E**) mean values at various simulated altitudes.

The tests revealed a decrease in blood saturation (between conditions F(1.197, 7.183) = 29.77; *p* = 0.0007)), and these changes were adequate and correlated with the reduction in oxygen concentration, which occurred with the increase in the simulated altitude (Figure 3).

#### Experiment 2

**Figure 4:**
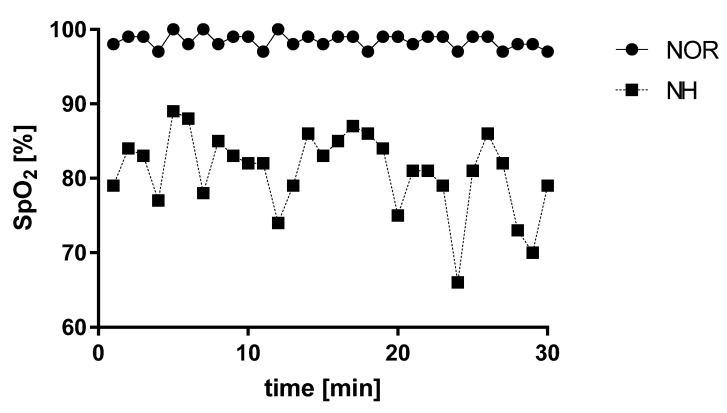
Effect of acute normobaric hypoxia exposure at FIO2 = 13% on blood saturation. Data are shown as the mean for the whole experimental group. NOR—normoxia; NH—normobaric hypoxia.

The saturation measurement at simulated 3500 m a.s.l. (FIO_2_ = 13%) for an enlarged experimental group decreased (*t* = 18.5; *p* < 0.0001; Cohen’s *d* = 1.186634), and it is shown in Figure 4.

## 4. Discussion

In the present study, acute moderate NH led to a decline in post-exposure cognitive performance. In contrast to our initial hypothesis, the hypoxic magnitude did not affect differentially post-exposure cognitive functions. 

It has been shown that a decrease in reaction time occurred at a simulated altitude of 3600 m a.s.l. and above [27]. Moreover, Kourtidou-Papadeli et al. (2008) showed that 16 min exposure at simulated 8000 ft (approx. 2440 m a.s.l.) was enough to increase error rate and decrease tracking performance [28]. 

The deterioration in human cognitive function in response to NH has been observed in previous studies. However, cognitive testing was carried out during NH exposure [27,28,41,42]. We observed a decline in cognitive performance after 30 min exposure to NH at simulated 3500 m (FIO_2_ = 13%). Even if the oxygen level in the mixture was lowered to 10% O_2_, a decline in cognitive functions was observed [2,27]. 

Based on our results, we can only speculate on the lack of potential impact of hypoxia on increased CBF, which could compensate/eliminate the destructive effect of hypoxia [22], since we did not measure it directly.

Moreover, we observe a post NH exposure decline in ST naming conditions. Color naming is presumed to be a more controlled response than reading. In the incongruent conditions in which the controlled naming response must be selected over the more habitual reading response, response should be slower [43]. In the Stroop paradigm, inhibition is thought to prevent the allocation of attention to the irrelevant stimulus dimension, allowing the participants to focus on the relevant dimension (i.e., not the name of the word, but the color of the ink in which the word is written) [43]. A decline in inhibitory control would therefore produce greater Stroop interference. This explanation is consistent with both behavioral and electrophysiological findings [44]. According to Bugg et al. (2007), the decline in naming differs depending on age [43]; however, it is not relevant in the age group that has been tested in our study [45]. It is possible that the NH exposure and thus desaturation contributed to these changes, as has also been shown in a recent study [46].

In the first experiment, we observed a tendency indicating a cognitive ability to decline in response to NH, but we did not observe any changes between the consecutive stages of hypoxia and executive function. Furthermore, the lack of difference between the executive function performance and hypoxia severity may arise from the cognitive testing time. Cognitive testing was carried out immediately after 30 min of exposure to hypoxia, under normoxic conditions, where the observed level of blood saturation corresponded to NOR. Since we did not notice significant differences between the various stages of hypoxia, for safety reasons, we decided to increase participant enlargement in the NH (simulated altitude 3500 m a.s.l) group (Experiment 2). After increasing the number of subjects, we observed a significant executive function impairment as a result of NH exposure.

However, attention should be paid to the previously described large individual response to hypoxia exposure [47,48] and the small sample of participants in the first phase of the study (Experiment 1).

Furthermore, in our study, cognitive tests were performed immediately after hypoxic exposure, where we could expect the reperfusion effect and thus improvement of cognitive functions, as has already been described in animal models [49] as well as in humans [50,51,52,53]. The mechanism of post-exposure cognitive improvement could be a result of increased nervous tissue supply in oxygen, energy, and/or hormones as a result of increased blood flow [54,55]. Furthermore, the moderate oxidative stress and inflammation induced by hypoxic exposure could stimulate/modulate the synthesis and/or release of BDNF and prevent hippocampal impairment [31,32,56]. Despite the lack of similar results in the literature on the executive functions following NH exposure, it seems that the changes in reaction time are associated with increased growth factor production, including upregulation of BDNF, which plays a critical role in sustaining memory function [57]. Hypoxia-sensitive genes regulate hypoxia-inducible factor 1, which regulates BDNF. Hypoxia-induced BDNF production can facilitate memory function by improving synaptic strength.

A few limitations of this study must be mentioned. The small sample size could have disturbed detection of significant differences in Experiment 1 group. Secondly, the study involved a specific group that could be enlarged to evaluate, e.g., age or sex difference. Thirdly, the cerebral blood flow and BDNF concentration were not measured. Thus, further investigations are needed.

In conclusion, the current findings indicate that NH exposure impairs cognition among healthy, young, physically active men even if cognitive tests are performed in NOR conditions immediately after exposure. 

## Figures and Tables

**Table 1:** Anthropometric characteristics of the participants.

*N* = 15	X	SD
Age [years]	23.1	2.1
Height [cm]	181	2.7
Weight [kg]	76.7	1.5
FAT [%]	13.9	1.4
FAT [kg]	11.2	1.6
FFM [kg]	67.1	1.5
BMI [kg∙m^−2^]	22.8	0.9

X—mean average; SD—standard deviation; FAT—adipose tissue; FFM—free fat mass or lean body mass; BMI—body mass index.
